# “Psychometric properties of the Norwegian foot function index revised short form”

**DOI:** 10.1186/s12891-022-05374-x

**Published:** 2022-05-03

**Authors:** Marianne Mørk, Aasne Fenne Hoksrud, Helene Lundgaard Soberg, Manuela Zucknick, Marte Heide, Karen Synne Groven, Cecilie Røe

**Affiliations:** 1 Department of Physical Medicine and Rehabilitation, Oslo University Hospital, Ullevål, Postboks 4956, Nydalen, 0242 Oslo, Norway; 2 Institute of Clinical Medicine, Faculty of Medicine, University of Oslo, Postboks 1078, Blindern, 0316 Oslo, Norway; 3 Research and Communication Unit for Musculoskeletal Health (FORMI), Division of Clinical Neuroscience, Oslo University Hospital, Postboks 4956, Nydalen, 0424 Oslo, Norway; 4 Norwegian Olympic and Paralympics Committee and Confederation of Sports, Postboks 5000, 0840 Oslo, Norway; 5 Department of Physiotherapy, Oslo Metropolitan University, Postboks 4, St. Olavs plass, 0130 Oslo, Norway; 6 Institute of Basic Medical Sciences, Faculty of Medicine, University of Oslo, Postboks 1110, Blindern, 0317 Oslo, Norway

**Keywords:** PROMs, Cross-cultural adaption, Psychometric properties, Foot disorders, Plantar fasciopathy, FFI-RS, Norwegian version

## Abstract

**Background:**

Foot disorders affect up to one quarter of the adult population. Plantar fasciopathy is a common cause of foot pain associated with decreased activity level and quality of life. Patient-reported outcome measures are important in assessing the burden of a condition as well as in research on the effects of interventions. The Foot Function Index revised short form (FFI-RS) is a region specific questionnaire frequently used in research. This study aimed to cross-culturally adapt the FFI-RS into Norwegian and to test its psychometric properties.

**Methods:**

The FFI-RS was translated into Norwegian (FFI-RSN) following international guidelines. 139 patients with foot disorders (88% with plantar fasciopathy) were included at baseline to measure internal consistency, explorative factor analysis, construct validity and floor and ceiling effects. 54 patients were included after 1 week for test-retest reliability and smallest detectable change analyses. 100 patients were included for responsiveness and minimal important change at 3 months.

**Results:**

Cronbach’s alpha for internal consistency was 0.97 and factor analysis supported the use of the total score of the FFI-RSN. Two out of three predefined hypotheses were confirmed by assessing the construct validity with Spearman’s correlation coefficient. Quadratic weighted Kappa for test-retest reliability showed 0.91 (95% CI 0.86–0.96) and the smallest detectable change was 6.5%. The minimal important change was 8.4% and the area under the receiver operating characteristic curve for responsiveness was 0.78 (95% CI 0.69–0.87). We found no floor or ceiling effects on the total score of the FFI-RSN.

**Conclusions:**

The present study showed excellent reliability of the FFI-RSN and supports the use of the total score of the questionnaire. Furthermore, we found the FFI-RSN to have acceptable responsiveness in relation to change in general health. Smallest detectable change, minimal important change and responsiveness were presented as novel results of the total score of the FFI-RS. FFI-RSN can be used to evaluate global foot health in clinical or research settings with Norwegian patients suffering from plantar fasciopathy.

**Trial registration:**

Clinical Trials.gov NCT04207164. Initial release 01.11.19.

## Background

Foot disorders are common and about 15–25% of the adult population experience foot pain that may affect their gait, balance and functional activities [1, 2]. The prevalence of foot pain increases with age and obesity (Body Mass Index > 30) and is more frequent among females. After adjusting for gender, age and BMI, patients with foot pain still score low on health related quality of life measures [2]. Plantar fasciopathy is a common foot disorder, although the prevalence is unclear, it is estimated at around 7%. This condition affects active and inactive persons and both genders equally [3]. Symptoms of depression, anxiety and stress are significantly associated with plantar fasciopathy, and in turn with poorer treatment outcome [4, 5].

Patient-reported outcome measures (PROMs) are used to collect information on perceived health directly from the patient. There is growing interest in patient-centred health-care systems, and PROMs are important measurement tools for capturing patients’ opinions on the impact and burden of their conditions [6]. We depend on valid, reliable and responsive outcome measures to map the characteristics of a patient group or estimate the effect of treatment. Reliability refers to the degree to which the PROM is free from measurement error. Validity indicates whether the PROM measures the intended constructs. Minimal important change (MIC) is the minimal change in score which the patients consider to be important. Lastly, a PROM needs to detect real change over time, the longitudinal validity or responsiveness [7].

Various PROMs are used to measure foot health. While some PROMs are disease specific [8], others are region specific like the Foot Function Index (FFI) [9] . In a review from 2020, Hijji et al. identified 25 different foot and ankle specific outcome measurement tools [10]. The FFI was used in 9% of the studies they described. In a Clinical Practice Guideline from 2014, Martin et al. advised clinicians to use FFI to evaluate the effect of interventions for patients with plantar fasciopathy [11]. The psychometric properties of FFI have been validated [12, 13]. The first version of the FFI, developed by Budman-Mak, Conrad and Roach in 1991 [9], faced criticism for not covering psychosocial aspects and quality of life related to foot health. The FFI was revised into a long (FFI-RL) and a short version (FFI-RS) by developing a theoretical model of foot function based on the World Health Organization International Classification of Function (ICF) [12]. As far as we are aware, the original FFI has been translated into seven languages [13], whereas the FFI-RL has only been translated into Brazilian Portuguese [14] and Turkish [15] and the FFI-RS into Polish [16]. The reliability, content and construct validity, effect size and the smallest detectable change (SDC) have been reported for the FFI-RL [13, 17, 18]. The FFI-RS has similar psychometric properties to the FFI-RL for internal consistency [12] and good test-retest reliability [16]. The responsiveness of the FFI-RS has only been reported on each subscale [19], and we are unable to find studies that report on the SDC, MIC or responsiveness of the total score of the FFI-RS [20]. We consider the FFI-RS to be more user-friendly as it is less burdensome for patients. However, a reliable, valid and responsive PROM specific to foot disorders such as the FFI-RS is, to our knowledge, not available in Norwegian. The present study will create a psychometric tested Norwegian foot specific questionnaire and contribute to diminishing the knowledge gaps surrounding the FFI-RS.

The aims of the present study were to cross- culturally adapt the FFI-RS into Norwegian (FFI-RSN) and to evaluate the reliability, validity and responsiveness of the FFI-RSN in a patient group with foot disorders.

## Methods

### Design

Cross-cultural adaption and psychometric testing of FFI-RSN were conducted with a prospective observational design and in a specialist health service setting.

### Cross- cultural adaptation of the FFS-RS

The developers gave their permission to the cross-cultural adaption of the FFI-RS into Norwegian in March 2017. A team of specialists in physical medicine and rehabilitation, followed international guidelines from Beaton et al. for the translation [21]. The English version of the FFI-RS was translated into Norwegian by two independent translators, with Norwegian as their mother tongue. We synthesised the results which a professional translator and a native English speaker then back translated. Based on a consensus from some of the translators involved and all the previous translations and written reports from every stages, the pre-final Norwegian version of the FFI-RS (FFI-RSN) was established. Ten patients with foot disorders filled in the pre-final version and responded to both written and oral questions concerning the difficulties, relevance and comprehensibility of the FFI-RSN. We assessed the comments from these pilot participants to create the tested version of the FFI-RSN.

### Procedure

**Fig. 1:**
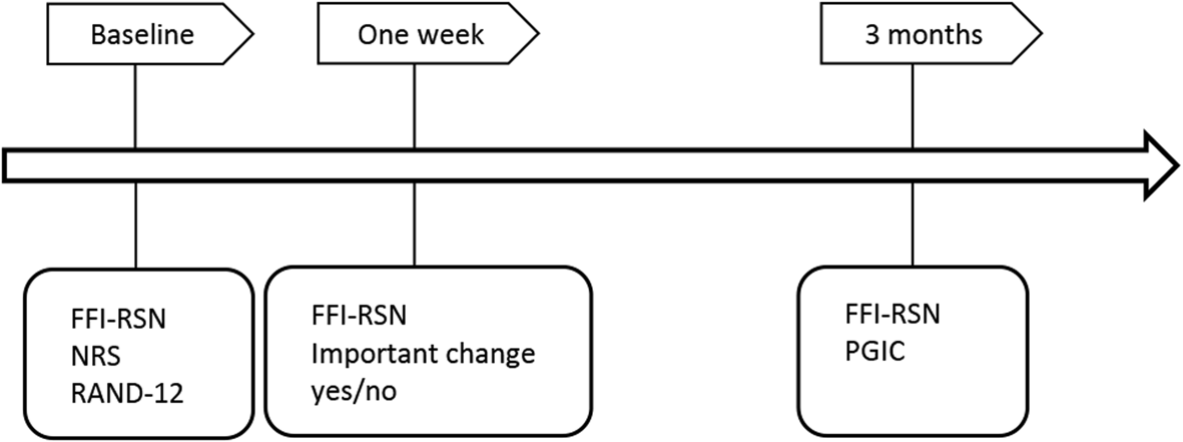
Timeline for procedure. FFI-RSN: Norwegian version of the Foot Function Index revised short form, NRS: Numeric Rating Scale for pain during activity and rest, RAND-12: Rand-12 Health Status Inventory, PGIC: Patient Global Impression of Change

The participants were assessed and completed questionnaires at baseline, 1 week later in the outpatient clinic and 3 months later by mail (Fig.1).

### Population

Patients referred to the Department of Physical Medicine and Rehabilitation at Oslo University Hospital (OUH) with foot disorders were assessed for eligibility. Inclusion criteria were age ≥ 18 years and foot complaints distal to the talocrural joint. Patients were excluded they had insufficient understanding of oral or written Norwegian. The majority of the participants (84%) were included through the ongoing randomized controlled trial with the aim of comparing the effectiveness of radial extracorporeal shock wave therapy (rESWT), sham-rESWTand a standardized exercise program with usual care for patients with plantar fasciopathy [22].

### Intervention

All patients received a thorough assessment off their foot problem by an experienced physician in physical medicine and rehabilitation at the outpatient clinic at OUH. Treatment was tailored to each individual and the patients had one to eight visits with an experienced physiotherapist. All patients received advice concerning coping strategies, and the majority 124 (89%), received customized foot orthoses. 30 (22%) patients were given radial extracorporeal shock wave therapy (rESWT) and 28 (20%) received sham rESWT. 51 (36%) patients were instructed in exercises. Due to the covid-19 pandemic, some visits were conducted as video or telephone consultations with advice and a progress update for those with exercise programs.

### Questionnaires

*Foot Function Index revised short form* (FFI-RS) was developed, according to Budiman-Mak et al., to obtain a total score for global assessment of foot function [12]. The FFI-RS comprises 34 questions concerning pain and stiffness, difficulty, activity limitation and social issues during the last week. Participants rate items on a four point Likert scale where 1 represents “no pain” and 4 “severe pain”. In addition, a fifth alternative, 5 “not applicable”, can be selected for six of the items. All item scores are summed (disregarding 5), divided by the individual’s highest possible score (based only on the applicable items) and multiplied by 100 [9]. The scores range between 0 and 100 with a lower score indicating better health.

*RAND-12 Health Status Inventory (RAND-12)* is the short version of RAND-36 and is a generic self-report PROM. It contains 12 questions related to eight different dimensions (physical functioning, role-physical, role-emotional, mental health, pain, vitality, social functioning and general health). Rand-12 results in a Mental Component Score (MCS-12) and a Physical Component Score (PCS-12). The scores range between 0 and 100 with a higher score indicating better health [23].

*Numeric Rating Scale (NRS)* is a self-report 11-point scale consisting of integers from 0 to 10 measuring for example pain intensity, where 0 represents “no pain” and 10 “worst imaginable pain”. Patients included in the present study scored pain intensity for both activity and rest during the last week. NRS is considered a reliable and responsive scale for registering pain [24].

*Patient Global Impression of Change (PGIC)* is a self-reported change in general health status with a 7-point Likert response scale where 1 is “very much improved”, 4 is “unchanged” and 7 is “very much worse” [25]. The wording in the present study was: “Compared to the beginning of the study, how is your general health status today?”

*Physical activity level* was assessed with the question: “What is your level of activity with respect to exercise/movement/physical exertion in your leisure time?” The four response categories were: 1. My leisure activity consists mostly of reading, watching TV or other sedentary hobbies. 2. I take walks, bike or exercise one way or another for at least 4 hours a week (including walking/biking to work, Sunday walks etc.). 3. I do recreational sports, heavy yard work or other similar activities for at least 4 hours per week. 4. I engage in strenuous training or competitive sports, regularly or several times a week.

### Statistical analysis

The statistical analyses were performed with IBM SPSS Statistics version 27 and R version 4.0.4 [26]. A significance level of *p* < 0.05 was applied and mean (standard deviation), median (interquartile range) and frequency (%) were reported. Sample size, choice of analysis and terminology were based on recommendations by the Consensus-based Standards for selection of health Measurement Instruments, COSMIN [27].

### Scoring FFI-RSN

All item scores (except for 5, “not applicable”) of the FFI-RSN were rescaled to a 0–3 range before being summed up. The sum was divided by the highest possible sum (excluding the 5 s) and converted into a 0–100% sum score scale.

### Reliability

The internal consistency of the FFI-RSN was evaluated using Cronbach’s alpha, with values > 0.7 considered good [28]. Dimensionality was tested with explorative factor analysis (minchi), guided by parallel analysis to find the number of factors. Oblimin rotation transformation was performed and loadings larger than 0.3 were used. The root mean square of the residuals (RMSR) and the Tucker-Lewis Index (TLI) were used to analyze the model fitting statistics. TLI of factoring reliability is considered good if > 0.95 and on the RMSR a value < 0.08 is regarded a good fit [29]. We used the psych and GPA rotation packages in R to implement the analyses described.

Test-retest reliability of the FFI-RSN was analysed using quadratic weighted Kappa and values > 0.8 were considered good test-retest reliability [28]. Quadratic weighted Kappa penalizes distant categories more than adjacent ones and is considered equal to Intraclass correlation coefficient (ICC_agreement_) [30].

The Standard Deviation (SD) and the reliability score from test-retest calculation were used to find Standard Error of the Measurement (SEM_agreement_): SD $\sqrt{1}-\mathrm{reliabilitet}$. The Smallest Detectable Change (SDC_individual_) was calculated as: SEM × 1.96 √2 [28].

### Validity

Construct validity was explored by the association between the FFI-RSN, pain and general health status. Hypotheses concerning the correlation between FFI-RSN, RAND-12 and NRS pain were defined prior to the analysis (Table 4).

### Minimal important change (MIC)

We used the Receiver Operating Characteristic curve (ROC) to calculate the MIC. Changes in FFI-RSN scores in patients improved by the anchor (PGIC) (sensitivity) and those not improved by the anchor (specificity) were calculated. The ROC curve was made by plotting sensitivity vs.1-specificity, where the highest combination of sensitivity and specificity was considered the MIC [31].

### Responsiveness

To determine the responsiveness of the FFI-RSN, we used the area under the ROC curve (AUC). PGIC was utilized as the anchor and “gold standard” in order to differentiate between those who were considered to be improved and those who were not. An AUC of at least 0.70 was considered acceptable responsiveness [28].

### Floor and ceiling effects

Floor and ceiling effects were calculated and recognized as present if more than 15% of the subjects achieved the lowest (0–10%) or highest possible total score (90–100%) [28].

### Missing items and exclusion

Missing items in the FFI-RSN were imputed with values based on Predictive Mean Matching (PMM) [32]. Patients were excluded if more than 25% of the items on the FFI-RSN were missing. We also excluded patients with any missing items in RAND-12, NRS and PGIC.

## Results

### Cross-cultural adaption of the FFI-RSN

Forward and backwards translation of the FFI-RSN was completed as planned with only minor discrepancies. One phrase in particular, “feeling awful”, was discussed among the translators, resulting in the use of a word more associated with feeling worried. References to the imperial system were converted to European metric units. After pretesting, we changed the layout due to difficulties identifying the “not applicable” responses. We sent the FFI-RSN to the developer Budiman-Mak. We chose to keep the original name of the questionnaire and added N for Norwegian version (FFI-RSN).

### Population

**Fig. 2:**
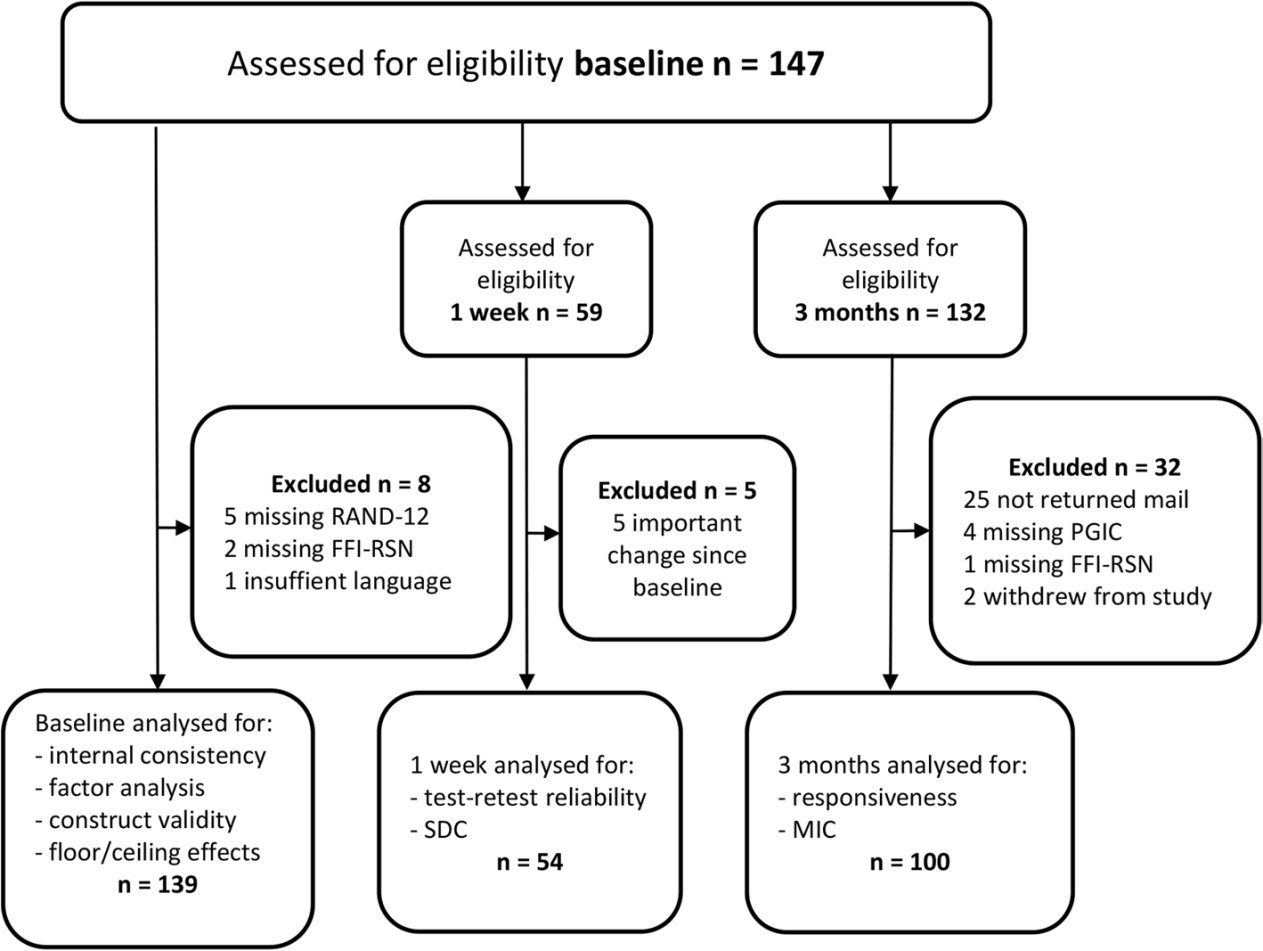
Flowchart for inclusion and analyses. RAND-12: Rand-12 Health Status Inventory, FFI-RSN: Norwegian version of Foot Function Index revised short form, PGIC: Patient Global Impression of Change, SDC: Smallest Detectable Change, MIC: Minimal Important Change

From March 2018 to November 2020, we assessed 147 patients with foot disorders for eligibility in the present study. Of the 139 included foot patients, 122 had plantar fasciopathy and the remaining 17 had other foot diagnoses. Figure 2 shows the inclusion procedures for the three cohorts and psychometric tests.

A total of 139 patients participated in the study, 54 were included in the test-retest analysis and 100 in the 3-month analyses (Table 1).

**Table 1:** Participant characteristics at baseline, one week and 3 months

Baseline characteristics	Baseline *n* = 139	One week *n* = 54	3 mnd. *n* = 100
Age in years^a^	45 (11)	45 (11)	47 (11)
Gender female^b^	109 (78)	40 (74)	81 (81)
BMI^a^	28 (5)	28 (5)	28 (5)
Duration symptoms^b^			
< 3 months	1 (0.7)	0	1 (1)
3–6 months	32 (23)	12 (22)	22 (22)
6–12 months	39 (28)	19 (35)	30 (30)
> 12 months	67 (48)	23 (43)	48 (48)
Physical activity level^b^	(*n* = 136)	(*n* = 53)	(*n* = 97)
Sedentary	23 (17)	11 (21)	16 (17)
Walking, biking ≥4 h/w	79 (58)	26 (49)	57 (59)
Recreational sport ≥4 h/w	28 (21)	14 (26)	20 (21)
Exercise / competition	6 (4)	2 (4)	4 (4)
NRS pain in activity^c^	6 (5–8)	6 (4–8)	6 (5–8)
NRS pain at rest^c^	3 (2–5)	3 (1–5)	3 (2–5)
RAND-12 sum score			*n* = 97
PCS12^c^	39 (32–46)	40 (33–48)	40 (34–48)
RAND-12 sum score			*n* = 97
MCS12^c^	43 (34–53)	46 (35–56)	46 (36–54)
FFI-RSN sum score^c^	44 (31–58)	40 (25–54)	43 (30–55)

^a^: mean (standard deviation), ^b^: frequency (%), ^c^: median (interquartile range). *n* numbers of participants, *BMI* Body Mass Index, *NRS* Numeric Rating Scale, *RAND12* Rand-12 Health Status Inventory, *PCS12* Physical Component Score, *MCS12* Mental Component Score, *FFI-RSN* Norwegian version of Foot Function Index revised short form

### Reliability

Test-retest reliability for the total sum of the FFI-RSN was estimated to 0.91 (95% CI 0.86–0.96). SD was 7.83 while the SEM_(agreement)_ was calculated as 2.35. SDC_(individual)_ was estimated to 6.53%.

Cronbach’s alpha for internal consistency of the FFI-RSN was estimated to 0.97 (95% CI 0.92–0 .98).

Parallel analysis and factor analysis supported 4 factors; 1. Difficulty and social issues, 2. Stiffness and social issues, 3. Activity limitation and social issues and 4. Pain and difficulty. Factor loadings and correlations are shown in Tables 2 and 3.

**Table 2:** Factor loadings

	Factor 4	Factor 2	Factor 3	Factor 1
Sum of squared loadings	6.78	6.15	3.95	2.75
Proportion variance	0.20	0.18	0.12	0.08
Cumulative variance	0.20	0.38	0.50	0.57
Proportion explained	0.35	0.32	0.20	0.13
Cumulative Proportion	0.35	0.66	0.87	1.00

**Table 3:** Factor correlation

	Factor 4	Factor 2	Factor 3	Factor 1
Factor 4	1.00	0.54	0.45	0.32
Factor 2	0.54	1.00	0.33	0.15
Factor 3	0.45	0.33	1.00	0.26
Factor 1	0.32	0.15	0.26	1.00

One question loaded less than 0.3 on all factors, “embarrassment due to footwear”. RMSR was 0.07 (acceptable), while the TLI of factoring reliability estimated to 0.10 (very low).

### Validity

Two predefined hypotheses were confirmed for construct validity for the FFI-RSN (Table 4). We found moderate negative correlation between FFI-RSN and Rand-12 Mental Component Score and a moderate positive correlation between FFI-RSN and Pain in activity measured with NRS. One hypothesis was not accepted; the moderate negative correlation between The FFI-RSN and RAND-12 Physical Component Score, which showed a negative correlation of 0.74.

**Table 4:** Results for testing of the construct validity of the FFI-RSN

	Spearman’s correlation coefficient FFI-RSN	*P* value	Predefined hypothesis	Hypothesis verified
Rand-12 PCS12	−0.74^a^	≤ 0.001	−0.4- - 0.6 (mod. Neg. cor.)	no
Rand-12 MCS12	−0.58^a^	≤ 0.001	−0.4- - 0.6 (mod. Neg. cor.)	yes
NRS pain activity	0.60^a^	≤ 0.001	0.4–0.6 (mod. Pos. cor.)	yes

^a^ Correlation is significant at the 0.01 level (2-tailed). FFI-RSN: Norwegian version of Foot Function Index revised short form. *RAND12* Rand-12 Health Status Inventory, *PCS12* Physical Component Score, *MCS12* Mental Component Score, *NRS* Numeric Rating Scale, mod. Neg. cor.: moderate negative correlation, mod. Pos. cor.: moderate positive correlation

### Minimal important change (MIC)

A change of global health in the PGIC of 1 or 2 was regarded as a meaningful improvement and 3–7 as not improved in the ROC analysis of the MIC. The distribution of the responses in PGIC is shown in Table 5.

**Table 5:** PGIC

Nr. PGIC		% of responses
1	Very much improved	9
2	Much improved	32
3	Minimally improved	25
4	No change	27
5	Minimally worse	2
6	Much worse	5
7	Very much worse	0

*Nr* number, *PGIC* Patient Global Impression of Change

**Fig. 3:**
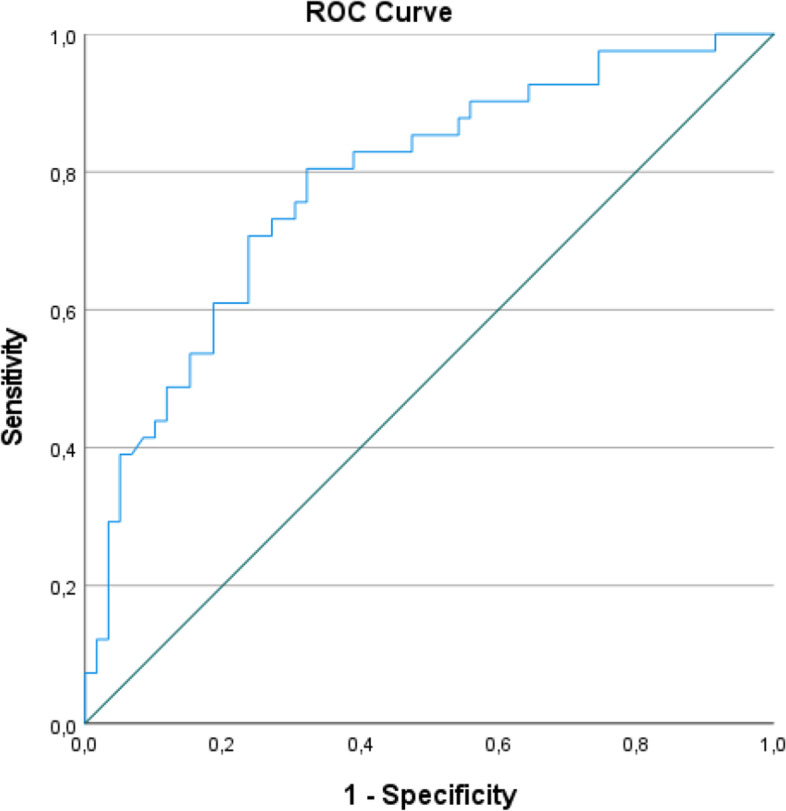
The Receiver Operating Characteristic (ROC) curve

We found one peak at 8.4% (sensitivity 0.8 and 1- specificity 0.3), as shown in Fig. 3. The analysis suggests that a change on the FFI-RSN ≥ 8.4% represents a meaningful improvement in general health for patients (MIC).

### Responsiveness

The AUC was estimated to 0.78 (95% CI 0.69–0.87).

### Floor and ceiling effects

We found no floor and ceiling effects on the total FFI-RSN at baseline. The total scores of the FFI-RSN showed that 4 respondents (3%) scored between 0 and 10% and 2 patients (1%) scored between 90 and 100%.

### Missing items and “not applicable”

We found 42 missing values in the FFI-RSN in the baseline cohort (1%). 14 missing items were found in the test-retest cohort (0.8%). 30 items were missing at 3 months (0.8%). These missing items were imputed. Missing items were randomly distributed at baseline and 1 week, but at 3 months the question “How much difficulty did your foot problems cause you when running?” was overrepresented (12%). Regarding the “not applicable” options, at baseline we found 319 (7%) responses, 137 at 1 week (7%) and 102 at 3 months (3%).

## Discussion

The results of the present study showed excellent reliability of the FFI-RSN and support the use of the sum score of this PROM. Furthermore, the responsiveness was acceptable. The SDC was 1.87 smaller than the MIC and two out of three predefined hypotheses were confirmed by measuring the construct validity.

The population in the present study consisted mainly of patients with plantar faciopathy, whereas the original FFI-RS was designed for and tested on people with arthritis [12]. We found the items of the FFI-RS to be relevant to our population, a conclusion which was supported by Budiman-Mak et al. who reported that rheumatoid arthritis and plantar fasciopathy were the two most frequent diagnoses where FFI and FFI-R were used in research. The two conditions were also reported as the most painful and disabling foot diagnoses in the study [13].

The results from the present study showed a very high Cronbach’s alpha for internal consistency for the FFI-RSN which is in line with results from other studies [12, 16]. This result implies that the multiple items in FFI-RSN have a high interrelatedness. Further the large number of items in the FFI-RSN contributes to high internal consistency. Factor analysis provides further information concerning the homogeneity of a PROM [33]. The results of the factor analysis and fitting statistic in the present study showed a very low factoring reliability. The four factors frequently overlapped, which was reflected in their high correlation and in the loading matrix. This finding supports those of Budiman-Mak et al., who developed the FFI-RS for use as a total foot functional score only [12].

Test-retest analyses in the present study imply good stability of the FFI-RSN which is in line with the results from the Polish version. The latter also tested the FFI-RS on a population that consisted mainly of women, but with rheumatoid arthritis [16].

We measured construct validity by calculating the correlation between the FFI-RSN and two generic PROMS. Considering that biopsychosocial aspects of health provided the theoretical foundation for the FFI-RS [12], we predicted moderate correlation between the FFI-RSN and both the physical and mental component scores of the RAND-12 as well as pain in activity. We found a higher correlation with the FFI-RSN and the physical dimensions of RAND-12, which indicates that FFI-RSN measures a higher degree of physical function than isolated pain and mental aspects. This finding should be taken into consideration when using FFI-RS.

The SDC in the present study indicates that, on an individual level, a change over 6.5% in the FFI-RSN is a real change and not a measurement error. To our knowledge, this is the first study reporting the SDC of FFI-RS. Rao et al. [17] found a SDC of 5% on the total score of the long version of the FFI-R which is in line with the results of the present study.

Our results imply that a change in the FFI-RSN larger than 8.4% is clinically important for patients. To the best of our knowledge, we found no other study presenting the MIC on the short version of the total score of the FFI-RS.

In the present sample, less than 50% of the participants improved according to our cutoff in the calculation of responsiveness at 3 months. As for other tendon conditions, patients with plantar fasciopathy take a long time to recover [34]. We could have measured the responsiveness with a longer time frame in order to obtain a larger improved group. However, when measuring responsiveness utilizing a retrospective global change question, a long period from baseline to follow-up, may contribute to increased recall bias [25].

Menz et al. [19] found medium to high responsiveness on the FFI-RS which is in line with the acceptable finding in the present study. It is difficult to compare the results from Menz et al. with our own because they only calculated responsiveness on the pain, stiffness and difficulty subscales of the FFI-RS. Their calculation of responsiveness was also conducted using a different method and in a different population than the present study’s.

The acceptable responsiveness means that the FFI-RSN is able to detect changes over time, making it applicable for use in the clinic and in trials measuring intervention effectiveness. However, the results of the present study need to be validated in different populations with various types of foot pain in order to strengthen the conclusion regarding invariance of the FFI-RSN.

Missing values were overrepresented in the question concerning difficulties when running. In the present population, 75% responded that they were either sedentary or walked/biked ≥4 hours a week. The question regarding running might not feel relevant to our sample and a solution could be to add a “not applicable” alternative to this question. We found no other study reporting how to handle missing values and the present study may serve as an example for future users of the FFI-RS.

We chose to rescale the FFI-RSN in order to get a true score from 0 to 100%. The scoring method of the original FFI with visual analogue scales (VAS) ranging from 0 to 100%, is described by Budiman-Mak et al. who added all item values together, divided by the total possible score and multiplied by 100 [9]. In the revised FFI (FFI-RL and FFI-RS) the scoring responses were converted into a four-point Likert scale, but the scoring method is, as far as we can see, not described specifically [13]. Other researchers have used the scoring method of the original FFI or only given a vague description of their scoring methods, and it is therefore difficult to interpret their results for comparison with our own [16, 17, 20, 35, 36]. We discussed the scoring of the FFI-RS with the developer and our own bio statistician who agreed that rescaling is one preferable option.

We found a low degree of ceiling and floor effect, which is in line with Rutkowski et al. who reported no such effects in the Polish validation of the FFI-RS [16]. The developer estimated a floor effect of 4.5% in their sample, which also supports the findings for the FFI-RSN [12].

### Strengths and limitations

A strength of the present study is that we have reported novel results on the psychometric properties of the FFI-RS. Furthermore, we have conducted a thorough forward and backword translation of the FFI-RSN with pilot testing. An additional strength is the presentation of a method for handling missing values and rescaling the score in order to obtain a score from 0 to 100% of the FFI-RS.

One limitation of the present study is that the population was exclusively recruited from a specialist setting at OUH, 78% in the baseline cohort were women and 88% had plantar fasciopathy. The results of the psychometric testing of the FFI-RSN are not necessarily generalizable to patient groups with other foot disorders and other settings. Furthermore, although the majority of patients with foot conditions in our department are diagnosed with plantar fasciopathy, we cannot exclude a sampling bias towards plantar fasciopathy due to the mentioned randomized controlled trial conducted in our department.

Rand-12 and NRS for pain, are to our knowledge, not validated for patients with foot diseases. Comparing the FFI-RSN to these two PROMs, and not to a gold standard when measuring construct validity, is therefore a limitation of the present study.

When measuring the MIC and responsiveness using PGIC, the participants in the present study answered a question concerning change in general health. When comparing the change in global aspects of life to foot specific aspects, the patients decide for themselves how to interpret the question, which could be a limitation. On the other hand, this method gives each patient the opportunity to choose what is most relevant to them, which can be considered a study strength [25]. A relatively large proportion (24%) of the participants did not return the 3-month questionnaires by mail which could also influence the results for responsiveness and MIC.

## Conclusions

The cross-cultural adaption and psychometric testing of the FFI-RSN as a total score in the present study showed excellent reliability. The SDC implies that a change of 6.5% or more on the FFI-RSN should be considered as a real change for individual patients and a change of 8.4% or more could be regarded as a meaningful improvement. The results of the present study contribute new and valuable psychometrics to the FFI-RS as well as a method for scoring and handling missing items. The FFI-RSN had satisfactory responsiveness and is applicable for use in clinical and research settings. Future studies should measure psychometric properties of the FFI-RSN in samples with greater variety of foot disorders, a larger proportion of males and in individuals with higher activity levels.

## Data Availability

The datasets used in the current study are available from the corresponding author upon reasonable request.

## Abbreviations

AUC: Area under the curve
COSMIN: Consensus-based standards for selecting of health measurement instruments
FFI: Foot Function Index
FFI-R: Foot Function Index revised form
FFI-RL: Foot Function Index revised long form
FFI-RS: Foot Function Index revised short form
FFI-RSN: Norwegian version Foot Functional Index revised short form
ICC: Intraclass correlation coefficient
ICF: International Classification of Function
MIC: Minimal important change
NRS: Numeric Rating Scale
OUH: Oslo University Hospital
PGIC: Patient Global Impression of Change
PMM: Predictive mean matching
PROMs: Patient-reported outcome measures
rESWT: Radial extracorporeal shock wave therapy
Rand-12: Rand-12 Health Status Inventory
MCS12: Mental Component Score
PCS-12: Physical Component Score
ROC: Receiver operating characteristics curve
RMSA: Root squared of the residuals
SD: Standard deviation
SDC: Smallest detectable change
SEM: Standard error of measurement
